# Mutual Interactions of Silymarin and Colon Microbiota in Healthy Young and Healthy Elder Subjects

**DOI:** 10.1002/mnfr.202400500

**Published:** 2024-10-30

**Authors:** Katerina Tomisova, Veronika Jarosova, Petr Marsik, Anna Mascellani Bergo, Ondrej Cinek, Lucie Hlinakova, Pavel Kloucek, Vaclav Janousek, Kateřina Valentová, Jaroslav Havlik

**Affiliations:** ^1^ Department of Food Science, Faculty of Agrobiology, Food and Natural Resources Czech University of Life Sciences Prague Kamycka 129 Prague Suchdol 165 00 Czech Republic; ^2^ Department of Pediatrics Charles University and University Hospital Motol V Uvalu 84 Prague 150 06 Czech Republic; ^3^ Biodviser Ltd 2 Pass St Oldham OL9 6HZ United Kingdom; ^4^ Institute of Microbiology of the Czech Academy of Sciences Videnska 1083 Prague 142 00 Czech Republic

**Keywords:** age‐related differences, gut microbiota, multi‐Omics analysis, polyphenols, silymarin metabolism

## Abstract

**Scope:**

This multi‐omic study investigates the bidirectional interactions between gut microbiota and silymarin metabolism, highlighting the differential effects across various age groups. Silymarin, the extract from *Silybum marianum* (milk thistle), is commonly used for its hepatoprotective effects.

**Methods and results:**

An in vitro fermentation colon model was used with microbiota from 20 stool samples obtained from healthy donors divided into two age groups. A combination of three analytical advanced techniques, namely proton nuclear magnetic resonance (^1^H NMR), next‐generation sequencing (NGS), and liquid chromatography–mass spectrometry (LC‐MS) was used to determine silymarin microbial metabolites over 24 h, overall metabolome, and microbiota composition. Silymarin at a low diet‐relevant dose of 50 µg mL^−1^ significantly altered gut microbiota metabolism, reducing short‐chain fatty acid (acetate, butyrate, propionate) production, glucose utilization, and increasing alpha‐diversity. Notably, the study reveals age‐related differences in silymarin catabolism. Healthy elderly donors (70–80 years) exhibited a significant increase in a specific catabolite associated with *Oscillibacter* sp., whereas healthy young donors (12–45 years) showed a faster breakdown of silymarin components, particularly isosilybin B, which is associated with higher abundance of *Faecalibacterium* and *Erysipelotrichaceae* UCG‐003.

**Conclusion:**

This study provides insights into microbiome functionality in metabolizing dietary flavonolignans, highlighting implications for age‐specific nutritional strategies, and advancing our understanding of dietary (poly)phenol metabolism.

## Graphical abstract

This study investigated gut microbiota and silymarin metabolism across age groups using an in vitro colon model. Silymarin at 50 µg mL^−1^ altered gut microbiota, reducing SCFAs production, glucose utilization, and increasing α‐diversity. Age differences were noted: elderly donors showed increased catabolite production linked to *Oscillibacter* sp., while young donors exhibit faster silymarin breakdown, associated with higher *Faecalibacterium* and *Erysipelotrichaceae* UCG‐003 abundance.

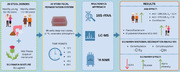

## 1 Introduction

Silymarin, an extract from fruits and seeds of the milk thistle *Silybum marianum* (L.) Gaertn., is basically a flavonolignan complex.^[^ ^1^, ^2^ ^]^ Although its composition may vary depending on the source, 70% is made up of the flavonolignans silybin A and B, isosilybin A and B, silychristin A and B, silydianin, and the flavanol taxifolin. Minor components include the flavonolignan isosilychristin and the corresponding 2,3‐dehydro derivatives of various flavonolignans, such as 2,3‐dehydrosilybin, 2,3‐dehydrosilychristin, and 2,3‐dehydrosilydianin.^[^ ^3^, ^4^ ^]^

Meta‐analyzes of clinical trials show that silymarin plays an important complementary role in the treatment of non‐alcoholic fatty liver disease, especially in terms of lowering levels of alanine and aspartate transaminases.^[^ ^5^, ^6^ ^]^

It has been shown to reduce hepatic fibrosis markers and serum levels of transforming growth factor beta‐1 (TGF‐β1), tumor necrosis factor alpha (TNF‐α), and interleukin 6 (IL‐6) in patients with chronic hepatitis B.^[^ ^6^ ^]^ Furthermore, it decreased total cholesterol and low‐density lipoprotein (LDL) levels while increasing high‐density lipoprotein (HDL) levels in hyperlipidemic individuals.^[^ ^7^ ^]^ The hepatoprotective effects may be attributed to influencing metabolic pathways to increase hepatic glutathione synthesis and cysteine availability, leading to higher antioxidant lipid defense in the liver^[^ ^8^ ^]^ or altering growth factor‐β‐mediated cell signaling pathways,^[^ ^9^ ^]^ as demonstrated by in vivo studies. In vitro studies suggest that silymarin may inhibit the proliferation of CD4^+^ cells, affect the pathways of nuclear factor kappa B (NF‐κB) activation^[^ ^10^ ^]^ or inhibit the production of pro‐inflammatory cytokines.^[^ ^11^ ^]^

The bioavailability of silymarin components after oral administration is in the range 20–50% due to low water solubility, extensive phase II metabolism, low permeability through intestinal epithelial cells, and involvement of efflux transporters.^[^ ^12^, ^13^, ^14^ ^]^ The flavonoids and flavonolignans undergo extensive enterohepatic circulation, with up to 80% of the absorbed silymarin constituents reintroduced into the intestine via bile.^[^ ^12^ ^]^ In the large intestine, these compounds are subject to extensive microbial metabolism. The biological activity of the parent compounds versus their microbial degradation products is currently a matter of contentious debate among experts.^[^ ^15^ ^]^

The ability of the colon microbiota to metabolize (poly)phenols varies considerably from person to person, as shown by previous studies, due to differences in microbiota composition.^[^ ^15^, ^16^ ^]^ This composition is influenced by numerous factors, including diet, general lifestyle, intake of probiotics and antibiotics, and environmental conditions.^[^ ^17^, ^18^, ^19^, ^20^ ^]^ Additionally, significant changes in microbiota composition have been observed in individuals over the age of 65 years.^[^ ^21^ ^]^ Consequently, the ability of the aging population to metabolize and utilize (poly)phenols and other dietary components may be compromised. From another perspective, recent studies have shown that dietary intake of (poly)phenols can alter the composition of the gut microbiota.^[^ ^22^ ^]^

Multi‐omic techniques have emerged as powerful tools in biomedical research, integrating data from multiple omics layers to provide a comprehensive understanding of complex biological systems. These approaches have advanced knowledge in personalized medicine and disease pathogenesis.^[^ ^23^, ^24^, ^25^ ^]^ The integration of metabolomics and sequencing has further deepened our understanding of the relationships between microbiota and metabolites.^[^ ^26^, ^27^, ^28^, ^29^, ^30^, ^31^, ^32^ ^]^

This study aims to elucidate the interactions between silymarin and the gut microbiome in aging populations using a multi‐omics approach (NGS, ^1^H NMR, and LC‐MS). By employing in vitro batch incubations with feces from 20 individuals, we examined age‐related effects and individual microbial profiles on silymarin catabolism and its impact on microbial composition and metabolic capacity. The goal was to understand these interactions to develop age‐specific nutritional strategies in relation to (poly)phenol intake.

## 2 Experimental Section

### 2.1 Study Design

Using a modified fecal fermentation system, silymarin was fermented in 96‐well deep well plates inoculated with feces from 20 human healthy donors. Samples of the fermentation liquid were collected at five time points (0, 2, 4, 8, and 24 h)^[^ ^33^, ^34^ ^]^ for metabolite profiling by LC‐MS and ^1^H NMR and assessment of microbial composition at 0 and 24 h by 16S rRNA sequencing. The effect of silymarin on the metabolite profile and composition of the gut microbiota was evaluated, as well as the effect of the donor's age on silymarin degradation.

### 2.2 Dosage Information

Silymarin (Batch 32621/M5) was obtained from Indena (Milan, Italy). Detailed quantitative analysis revealed a flavonolignan content of 62.81% w/w, with the most abundant components being silybin B (13.8%) and silybin A (9.3%), and the presence of a polymer fraction accounting for 36.6%.^[^ ^4^ ^]^ The stock solution was prepared at a concentration of 10 mg mL^−1^ in DMSO (Sigma‐Aldrich, Prague, Czech Republic) and kept at 4 °C. A working solution of concentration 2 mg mL^−1^ was prepared by mixing the stock solution with fermentation medium. The concentration of silymarin in each 2 mL well during the incubations was 50 µg mL^−1^.

### 2.3 Fecal Samples and Ethics Statement

Human fecal samples were collected from 20 volunteers. All subjects signed an informed consent form for inclusion prior to their participation in the study. The study was conducted in accordance with the Declaration of Helsinki and the protocol was approved by the ethics committee of the University Hospital Královské Vinohrady in Prague (LEK‐VP/01/0/2019). The donors were divided into two groups: healthy young (age 12–45 years) and healthy elders (age 70–80 years). The demographic data were shown in Table S1, Supporting Information. The donors had no dietary restrictions and did not suffer from digestive problems.

### 2.4 In Vitro Fecal Fermentation System

A high‐throughput method of ex vivo fermentation method was used, using a modification of a previous study^[^ ^33^ ^]^ adapted to polypropylene 96‐well deep square well plates with working volume 2.0 mL (VWR, Radnor, PA, USA). The experiment was conducted in batches organized by donors, with two donors processed per batch each day. A single independent experiment was performed.

#### 2.4.1 Media and Buffers

The fermentation medium used previously^[^ ^33^ ^]^ was prepared as a mixture of 450 mL of distilled water, 2.25 g of tryptone, 2.25 g of glucose, 1.125 g of maltose, 2.25 g of yeast extract, 50.7 µL of vitamin K1 (0.5 mg L^−1^), and 5.07 mg haemin (5 mg L^−1^), 112.5 µL of a micromineral solution (2.5 g CaCl_2_, 2.5 g MnCl_2_·4H_2_O, 0.25 g CoCl_2_·6H_2_O, 1.25 g FeCl_3_, and distilled water up to 25 mL), 225 mL of a macromineral solution (5.7 g Na_2_HPO_4_, 6.2 g KH_2_PO_4_, 0.3 g MgSO_4_, and distilled water up to 1 L), 225 mL of CO_3_ buffer (4 g NH_4_HCO_3_, 35 g NaHCO_3_, and distilled water up to 1 L) and 1.125 µL of 0.1% resazurin solution. All chemicals were obtained from Merck (Darmstadt, Germany).

The sodium phosphate buffer for preparation of the fecal slurries was made up of 70 mM KH_2_PO_4_ and 70 mM Na_2_HPO_4_ (pH 7 at 20 °C). The reducing solution consisted of 312.5 mg of cysteine hydrochloride, 2 mL of 1 M NaOH, 101.5 mg of Na_2_S, and 50 mL of distilled water.

#### 2.4.2 Preparation of 96‐Well Deep Well Plates

The freshly prepared fermentation medium was boiled for 7 min using a cotton cup and cooled to approximately 37 °C while being purged with oxygen‐free nitrogen gas. The pH of the medium was adjusted to pH 7.0 with 6 M HCl.

The wells were filled with 835 µL of fermentation medium and 40 µL of reducing solution. The plates were then sealed in vacuum bags together with GENbag anaer (Biomérieux, Lyon, France) and stored at 4 °C until the next day to establish anaerobiosis.

#### 2.4.3 Collection of Feces

All donors collected feces independently using a collection kit. Samples were collected in a 1 L plastic container, sealed in a plastic bag containing GENbag anaer and stored for a maximum of 2 h. Fresh feces were homogenized in a stomacher bag (Laboratory Blender, Stomacher 400 Circulator, EU) with sodium phosphate buffer for 30 s and the resulting 24% fecal slurry was filtered through a nylon mesh.

#### 2.4.4 Fermentation

The plates prepared on the previous day were heated to 37 °C and then 100 µL of the fecal suspension (sodium phosphate buffer as a control) and 25 µL of the silymarin working solution (DMSO as a control) were added. The final concentration of the tested extract in the solution was thus 50 µg mL^−1^. The plates were sealed in vacuum bags with GENbag anaer and incubated at 37 °C on a shaker (100 rpm). At time points 0, 2, 4, 8, and 24 h, one plate was removed from the experiment and 950 µL of each well was aspirated in microtubes containing 50 µL of sodium azide (the final concentration of NaN_3_ in the samples was 1.5 mg mL^−1^) to stop the fermentation process. The fermentation liquids were stored at –80 °C until final analysis.

### 2.5 ^1^H NMR Metabolomics

Analyses were conducted by a modification of a previous study.^[^ ^35^ ^]^

#### 2.5.1 Sample Preparation

Fermentation liquid stored at –80 °C was thawed at room temperature, mixed with a vortex oscillator and centrifuged (4 °C, 24 400 × *g*, 10 min). Then, 600 µL of supernatant was mixed with 67 µL of NMR buffered solution [1.5 M K_2_HPO_4_/1.5 M NaH_2_PO_4_, pH 7.4, 0.2% NaN_3_ and 5 mM 3‐(trimethylsilyl)propionic‐2,2,3,3‐*d*_4_ acid sodium salt in D_2_O], and centrifuged again (4 °C, 24 400 × *g*, 5 min). Then, 600 µL of the supernatant was transferred to 5‐mm NMR tubes (NORELL Inc., Morganton, NC, USA).

#### 2.5.2 Spectra Acquisition

^1^H NMR spectra were acquired using a Bruker Avance III HD spectrometer equipped with a broad band fluorine observation (BBFO) SmartProbe with *z*‐axis gradients (Bruker BioSpin, Billerica, MA, USA) operating at a proton frequency of 500.23 MHz. The ^1^H NMR spectra were recorded using Topspin 3.57 software (Bruker BioSpin) and the Bruker pulse sequence “noesypr1d” for one‐dimensional nuclear Overhauser enhancement spectroscopy. Presaturation was applied to suppress the water signal at 4.704 ppm. The temperature was set to 298 K (25 °C) and the following acquisition parameters were used: 64 scans, 64k data points with a spectral width of 16 ppm, relaxation delay of 1 s, acquisition time of 4 s, and mixing time of 0.1 s. Tuning, locking, shimming, and 90° pulse calibration optimization were performed for each sample using the Bruker standard routine. The free induction decay (FID) signals recorded were multiplied by a 0.3 Hz line broadening function prior to Fourier transformation.

#### 2.5.3 ^1^H NMR Data Processing

The FID signals were processed in MestReNova software (Version 14.1.0; Mestrelab Research S.L., Santiago de Compostela, Spain) and the ^1^H NMR spectra were manually phased, baseline corrected using the Whitaker smoother algorithm and the TSP signal was referenced at 0.0 ppm. The pre‐processed spectra were then exported as JCAMP files to Chenomx NMR Suite 8.5 software, Professional Edition (Chenomx Inc., Edmonton, AB, Canada), for annotation and quantification, identifying a total of 35 compounds (Table S2, Supporting Information). Concentrations of compounds not identified in the spectra were replaced with 1/3 of the lowest recorded concentration among all the spectra.

### 2.6 LC‐MS Analysis of Silymarin Catabolites

Analyses were conducted by a modification of a previous study.^[^ ^15^ ^]^

#### 2.6.1 Sample Preparation

Fermentation liquid stored at –80 °C was thawed at room temperature, mixed with methanol in a 1:1 ratio and refrigerated at –18 °C for 24 h to precipitate the proteins. After centrifugation (4 °C, 24 400 × *g*, 10 min), 50 µL of supernatant was mixed with 900 µL of solvent (50% methanol in water) and 50 µL of internal standard fluconazole at a final concentration of 0.1 µg mL^−1^. Samples were measured immediately after preparation.

#### 2.6.2 LC‐MS Data Acquisition

Analyses were performed using an ultrahigh‐performance LC‐MS system consisting of a Dionex Ultimate 5000 liquid chromatograph (Thermo Fisher Scientific, Waltham, MA, USA) and an ultrahigh‐resolution accurate‐mass (HRAM) quadrupole time‐of‐flight (Q‐ToF) mass spectrometer (IMPACT II; Bruker Daltonik, MA, USA). For chromatographic separation by gradient elution, an Acclaim RSLC 120 C18 column (2.2 µm, 2.1 × 100 mm; Thermo Fisher Scientific, Waltham, MA, USA) and a combination of mobile phases (Phase A, 0.1% formic acid; Phase B, methanol) were used. Gradient elution started at 2% Phase B (0–1 min) and continued at 100% Phase B after 18 min, followed by a wash (up to 22.5 min) and a 10‐min equilibration of 2% Phase A. The column temperature was controlled at 35 °C and the mobile phase flow rate was set at 250 µL min^−1^. The sample injection volume was 5 µL. Samples were measured by electrospray ionization in positive mode and spectra were acquired with a mass resolution of > 60 000 and a scan rate of 1 Hz over a mass‐to‐charge (*m/z*) ratio range of 60–1 500 *m/z*. Data were stored and processed in centroid mode.

#### 2.6.3 LC‐MS Data Processing

The data matrix was aligned, annotated, and filtered in XCMS^[^ ^36^ ^]^ using the default settings of the spectrometer used. For data filtering, the Wilcoxon signed rank test was employed independently for each time point to select features showing significantly different intensities between the controls and treatments across all donors. As a result, a data matrix containing intensity, *m/z* ratio, retention time (*t_R_*), fold change, and *p* values was created and manually curated to obtain a list of parent silymarin components and their plausible catabolites.

### 2.7 Microbiome Analysis

A modified method from a previous study was applied to the analysis.^[^ ^37^ ^]^ Samples were centrifuged at 4 °C and 5 000 × *g* for 10 min and DNA was extracted from the bacterial sediment using a DNeasy PowerSoil Kit (Qiagen, Hilden, Germany) according to the manufacturer's instructions. The quantity of DNA was determined by real‐time polymerase chain reaction (PCR) using Qiagen HotStarTaq DNA Polymerase and primers specific for the V4 region of the 16S rRNA gene, along with a probe. The V4 region of the 16S rRNA gene was amplified with specific primers^[^ ^38^ ^]^ containing adapters and indices for sample identification. Each sample was amplified in duplicate. The PCR reaction contained 18 µL of AccuPrime Pfx SuperMix (Invitrogen, Carlsbad, CA, USA), 1.2 µL of each primer (10 µM), and 1.2 µL of extracted DNA. The cycling conditions were as follows: initial denaturation at 95 °C for 5 min, 30 cycles at 95 °C for 15 s, at 55 °C for 30 s, and at 68 °C for 1 min, followed by a final step at 68 °C for 5 min. The amplified indexed DNA fragments were purified and brought to the same concentration using the SequalPrep Normalization Plate Kit (Applied Biosystems, Waltham, MA, USA). The amount of balanced PCR products was determined visually by gel electrophoresis. Samples were pooled and the concentration of each pool was measured using the Qubit dsDNA HS Assay Kit (Invitrogen). The final pool with approximately 2 nM concentration was sequenced on the Miseq platform (Illumina, San Diego, CA, USA) using the V2 kit of 2 × 250 bp.

### 2.8 Statistical Analysis

Samples were analyzed as single biological replicates for each donor, with all analyses performed in a single technical replicate. This approach was chosen because of the in vitro nature of the experiment and the minimal variation observed between replicates.

Selected statistical analyses were performed for area under the curve (AUC) values over 24 h and for this purpose the AUC was determined using the trapezoidal method. Other analyses were performed separately for each time point. To test the effect of the overall change in metabolite levels, a non‐parametric permutational multivariate analysis of variance (PERMANOVA) was performed with the R package “vegan” (Version 2.6‐4) using the adonis2 method with 9999 permutations.^[^ ^39^ ^]^ The Bray–Curtis distance was used to calculate a dissimilarity matrix.

To test the effect of the treatment and age on levels of metabolites, the paired Student's *t*‐test (effect of treatment) and independent Student's *t*‐test (effect of age) were employed, respectively, reflecting the correlation structure of the data. Prior to these tests, a logarithmic transformation (base 10) was applied to the data. Given the presence of zero values within the LC‐MS datasets, a uniform increment was added to all measurements before applying the logarithm. In this study, a threshold of *p* < 0.05 was established for statistical significance. In addition, false discovery rates (FDRs) were performed for the metabolites within multiple comparisons at a significance level of *p* = 0.1, resulting in corresponding *q* values for each metabolite.

Principal component analysis (PCA) was applied to the ^1^H NMR metabolite profiles, focusing on the comparison of two categories: control versus treatment. Data preprocessing involved both centered log ratio and logarithmic transformations, using R packages phyloseq (Version 1.46.0)^[^ ^40^ ^]^ and microViz (Version 0.11.0)^[^ ^41^ ^]^ to perform the analysis and plot the results.

The α‐diversity indices were assessed according to species evenness (Shannon) and species diversity (Simpson) using the R package phyloseq^[^ ^40^ ^]^ and the difference between treatment and control groups was tested using the paired Student's *t*‐test.

NGS/operational taxonomic unit (OTUs) data, LC‐MS data of silymarin components and of the catabolites were integrated using multiblock sparse partial least‐squares discriminant analysis (sPLS‐DA), implemented as a Data Integration Analysis for Biomarker discovery using Latent cOmponents (DIABLO) method in the R package mixOmics (Version 6.26.0).^[^ ^42^, ^43^ ^]^ Visualization was provided by methods from the mixOmics package.^[^ ^44^ ^]^ First, the block.sPLS‐DA function was used to determine the optimal number of components based on the performance of the model when considering the centroid distance technique and the lowest balanced error rate, with a fivefold cross‐validation (repeated 100 times). The optimized model contains 30 OTUs, three silymarin components for LC‐MS and five silymarin microbial catabolites for LC‐MS. In addition, the plotDiablo function was used to generate a plot showing the overall correlation between the most discriminant amplicon sequence variants and catabolites, with a network function used to visualize correlations of >0.6.

## 3 Results

### 3.1 Characterization of Silymarin Complex

**Figure 1:**
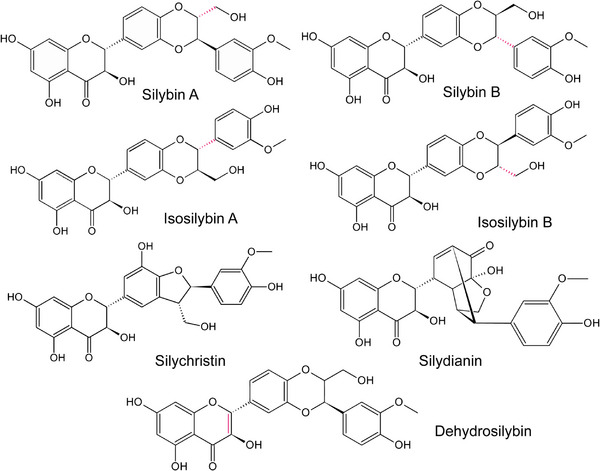
Molecular structure of silymarin flavonolignans.

LC‐MS analysis revealed eight individual silymarin components: silybin A, silybin B, isosilybin A, isosilybin B, silydianin, silychristin, an unknown flavonolignan (structural isomer with *m/z* 483.129) and 2,3‐dehydrosilybin (*m/z* 481.112). Interestingly, no taxifolin was detected in this fraction. A list of identified silymarin components and their relative abundances is provided in Table 1 and their molecular structures are illustrated in Figure 1. The most abundant constituent was silybin B, comprising 33.4% based on the integral of molecular ion, followed by silybin A (19.4%) and silydianin (19.2%).

**Table 1:** Identification and relative abundance of flavonolignans in silymarin mixture at 0 h by liquid chromatography–mass spectrometry (LC‐MS).

Name	*m/z* [M+H]^+^	*t_R_* [min]	Neutral formula	Calculated mass	Rel. % in the mixture^a)^
Silybin B	483.1287	12.26	C_25_H_22_O_10_	482.44	33.4
Silybin A	483.1288	12.07	C_25_H_22_O_10_	482.44	19.4
Silydianin	483.1287	10.94	C_25_H_22_O_10_	482.44	19.2
Isosilybin A	483.1287	12.69	C_25_H_22_O_10_	482.44	10.2
Silychristin	483.1287	10.53	C_25_H_22_O_10_	482.44	9.8
Isosilybin B	483.1287	12.71	C_25_H_22_O_10_	482.44	8.1
Unknown	483.1288	10.29	C_25_H_22_O_10_	482.44	1.9
2,3‐Dehydrosilybin	481.1133	10.80	C_25_H_20_O_10_	480.42	1.0

^a)^ Based on MS peak area.

### 3.2 Microbial Degradation of Silymarin

**Figure 2:**
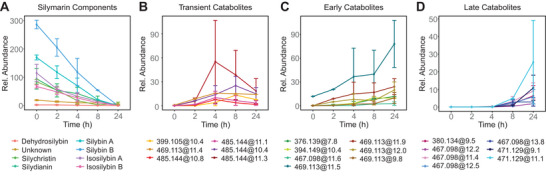
Time course of the metabolism of silymarin components and their degradative metabolites. The data represent mean values calculated across all donors (*n* = 20), each based on a single biological and technical replicate per donor. Error bars  =  SD, A) silymarin components; B) transient catabolites; C) final catabolites with an early increase; and D) final catabolites with a late increase.

The original silymarin components gradually degraded with time in the presence of the fecal microbiota but their proportions remained relatively constant. Complete degradation of all components by the fecal microbiota was observed after 24 h, with silychristin and 2,3‐dehydrosilybin being completely degraded after 8 h, as shown in Figure 2A. Twenty catabolites were formed from the original components during the fermentation. Of these, six were defined as transient, characterized by their maximum intensity at 4 or 8 h. The others were final catabolites (mainly structural isomers), with maximum intensity reached after 24 h. Notably, these final catabolites can be further classified as early catabolites when detected at 2 or 4 h of incubation and as late catabolites when detected after ≥8 h of incubation, as illustrated in Figure 2.

The catabolites were further investigated by classifying their relative intensity based on their AUC value over 24 h. For clarity, we have named the catabolites according to their *m/z* value and retention time, connected by “@.” The most abundant catabolite was a final catabolite with *m/z* 469.113 eluting at 11.5 min (469.113@11.5); this is likely to be a demethylation product of the most abundant silybin B, which is formed by the loss of 14.016 *m/z*. The other four metabolites with *m/z* 469.113 are likely to be demethylation products of less abundant silymarin flavonolignan isomers. In addition, the remaining final catabolites were likely formed by dihydroxylation, demethylation, or hydrogenation. Demethylation in combination with other transformations is mainly associated with the early catabolites, whereas the late catabolites are mainly the result of dehydroxylation. A comprehensive list of catabolites and their predicted chemical changes can be found in Table 2.

**Table 2:** Microbial catabolites of silymarin components identified by liquid chromatography–mass spectrometry (LC‐MS).

Name	*m/z* [M+H]^+^	*t_R_* [min]	*m/z* ^a)^	Neutral formula	Monoisotopic mass	Loss	Addition	Anticipated reactions	Relative AUC average intensity [%]^b)^
469.113@11.5	469.1128	11.53	−14.0162	C24H20O10	468.1056	CH3	H	Demethylation	100
485.144@11.3	485.1442	11.25	2.0152	C25H24O10	484.1369		2H	Hydrogenation	59.8
485.144@10.4	485.1442	10.36	2.0152	C25H24O10	484.1369		2H	Hydrogenation	35.8
469.113@11.9	469.1129	11.86	−14.0161	C24H20O10	468.1056	CH3	H	Demethylation	35.7
469.113@11.4	469.1128	11.38	−14.0162	C24H20O10	468.1056	CH3	H	Demethylation	27.7
469.113@12.0	469.1129	12.01	−14.0161	C24H20O10	468.1056	CH3	H	Demethylation	21.7
471.129@11.1	471.1285	11.13	−12.0005	C24H22O10	470.1213	CH3	3H	Demethylation; hydrogenation	21.2
399.105@10.4	399.1052	10.42	−84.0238	C25H18O5	398.1154			Complex of reactions	17.4
376.139@7.8	376.1391	7.84	−106.9899						15.7
394.149@10.4	394.1496	10.42	−88.9794						15.7
485.144@11.1	485.1442	11.07	2.0152	C25H24O10	484.1369		2H	Hydrogenation	10.6
469.113@9.8	469.1130	9.78	−14.016	C24H20O10	468.1056	CH3	H	Demethylation	9.3
467.098@11.4	467.0975	11.41	−16.0315	C25H22O9	466.1264	OH	H	Dehydroxylation	8.8
471.129@9.1	471.1286	9.11	−12.0004	C24H22O10	470.1213	CH3	3H	Demethylation; hydrogenation	8.1
485.144@10.8	485.1442	10.80	2.0152	C25H24O10	484.1369		2H	Hydrogenation	6.2
380.134@9.5	380.1341	9.58	−102.9949						5.9
467.098@12.5	467.0975	12.48	−16.0315	C25H22O9	466.1264	OH	H	Dehydroxylation	5.7
467.098@13.8	467.0975	13.77	−16.0315	C25H22O9	466.1264	OH	H	Dehydroxylation	4.3
467.098@11.6	467.0975	11.6	−16.0315	C25H22O9	466.1264	OH	H	Dehydroxylation	3.8
467.098@12.2	467.0975	12.24	−16.0315	C25H22O9	466.1264	OH	H	Dehydroxylation	1.6

^a)^ *m/z* represents the difference in mass‐to‐charge ratio (*m/z*) between the catabolites and the original silymarin flavonolignans with *m/z* 483.129.

^b)^ Relative area under the curve (AUC) average intensity is represented as the percentage of the most abundant catabolite 469.113@11.5 (*n* = 20).

The second most abundant catabolite overall was the transient compound 485.144@11.3, which had three other similarly transient isomers. We hypothesize that these are formed by hydrogenation of double bonds of silymarin flavonolignans.

Finally, for four of the catabolites identified we could not predict the structural changes and no further structural information could be extracted from the available data.

### 3.3 Effect of Silymarin on the Gut Metabolome

^1^H NMR metabolomic analysis provided insights into 35 abundant metabolites present in the fermentation liquid, including amino acids, carbohydrates, short‐chain fatty acids (SCFAs) and phenolic acids (Table S2, Supporting Information).

**Figure 3:**
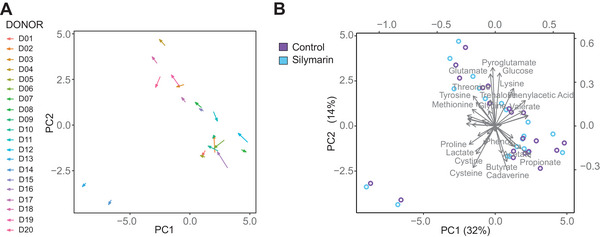
Principal component analysis of the metabolic profile of the medium by ^1^H NMR. The data represent mean values calculated across all donors (*n* = 20), each based on a single biological and technical replicate per donor. Error bars  =  SD A) Score plot. The arrows in the figure represent vectors depicting the direction of change between the control group and the silymarin‐treated group from the same donor. B) Corresponding loadings plot.

**Figure 4:**
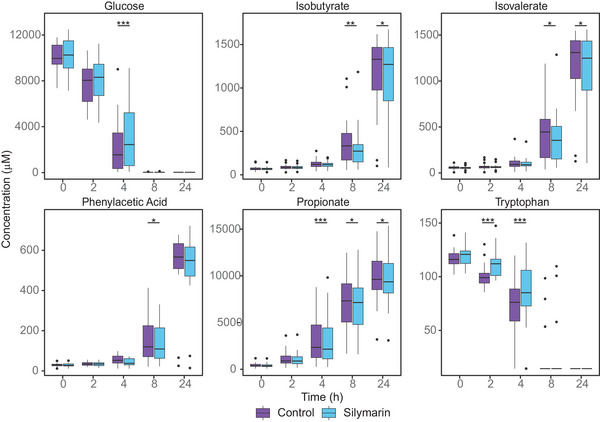
Time course of six most significant metabolites compared between silymarin treatment and control as total area under the curve (AUC) over 24 h, expressed as box plots for each time point. The data represent mean values calculated across all donors (*n* = 20), each based on a single biological and technical replicate per donor. Error bars = SD. Paired test of log‐transformed data: ^*^ *p* < 0.05; ^**^ *p* < 0.01; ^***^ *p* < 0.001.

PCA of the ^1^H NMR metabolite profiles, which compared silymarin with controls using the AUC values, did not reveal clear clustering. This lack of distinct grouping was primarily due to considerable variation among individual donors. The individual metabolomic profile is significantly more pronounced than the effect of silymarin treatment (Figure 3). Considering these findings, a pairwise statistical analysis was conducted for the overall donor population regardless of age (*n* = 20), which identified 14 metabolites that significantly differed with the addition of silymarin. The introduction of silymarin was linked to a notable decrease in the production of SCFAs such as acetate, butyrate, and propionate, as well as branched‐chain fatty acids (BCFAs) including isobutyrate and isovalerate. Additionally, a reduction was observed in certain carboxylic acids, specifically valeric acid and phenylacetic acid, as indicated by changes in the AUC. Furthermore, silymarin addition resulted in lower utilization of glucose, trehalose, and certain branched‐chain amino acids (BCAAs, i.e., valine, leucin, isoleucin), as evidenced by the higher AUC for these metabolites. Figure 4 illustrates the time course of the six most significantly different metabolites, with statistical differences for each time point. When this analysis was performed separately for each age group (*n* = 10), the effect of silymarin was similar in both groups to that observed in the overall donor population. However, the effect was more pronounced in healthy elder donors. In older subjects, nine metabolites showed differences with the addition of silymarin, whereas only seven metabolites were affected in younger donors. Additionally, the relative fold changes were higher in the older group compared to the younger group. Table 3 shows the effects of silymarin on metabolite changes in both age groups and the overall donor population, along with the respective fold changes.

**Table 3:** ^1^H NMR‐detected metabolites significant by paired *t*‐test of log‐transformed data between silymarin treatment and control as total area under the curve (AUC) over 24 h.

Metabolite	Healthy young + Healthy elders (*n* = 20)			Healthy young (*n* = 10)			Healthy elders (*n* = 10)		
	*p*	*q* (FDR)	Relative change [%]^a)^	*p*	*q* (FDR)	Relative change [%]^a)^	*p*	*q* (FDR)	Relative change [%]^a)^
Acetate	3.10 × 10^−3^	0.0136	−2.32	1.91 × 10^−2^	0.1572	−2.02	6.17 × 10^−2^	0.1659	−2.66
Butyrate	2.66 × 10^−3^	0.0133	−3.02	5.79 × 10^−2^	0.2026	−2.66	2.86 × 10^−2^	0.1081	−3.44
Glucose	1.76 × 10^−3^	0.0103	10.20	3.13 × 10^−2^	0.1572	12.16	1.40 × 10^−2^	0.0791	8.79
Isobutyrate	1.90 × 10^−4^	0.0022	−8.16	5.76 × 10^−2^	0.2026	−6.77	1.05 × 10^−3^	0.0178	−9.61
Isoleucine	4.38 × 10^−2^	0.1133	3.04	2.19 × 10^−1^	0.3835	1.30	1.16 × 10^−1^	0.2297	4.72
Isovalerate	3.69 × 10^−5^	0.0006	−8.73	3.31 × 10^−2^	0.1572	−6.16	1.59 × 10^−4^	0.0054	−11.41
Leucine	4.53 × 10^−2^	0.1133	2.64	3.35 × 10^−1^	0.4693	0.71	4.66 × 10^−2^	0.1585	4.45
Phenylacetic Acid	7.23 × 10^−4^	0.0052	−6.31	1.90 × 10^−2^	0.1572	−3.94	9.26 × 10^−3^	0.0773	−8.53
Phenylalanine	3.22 × 10^−2^	0.1023	2.83	2.85 × 10^−1^	0.4535	1.34	6.34 × 10^−2^	0.1659	4.28
Propionate	7.42 × 10^−4^	0.0052	−3.04	3.59 × 10^−2^	0.1572	−3.02	1.14 × 10^−2^	0.0773	−3.07
Trehalose	3.6 × 10^−3^	0.0140	4.83	2.76 × 10^−2^	0.1572	6.26	7.48 × 10^−2^	0.1818	3.38
Tryptophan	3.05 × 10^−6^	0.0001	8.89	1.10 × 10^−3^	0.0384	8.17	1.83 × 10^−3^	0.0207	9.64
Valerate	6.83 × 10^−3^	0.0239	−4.19	1.29 × 10^−1^	0.3237	−2.09	2.50 × 10^−2^	0.1063	−6.35
Valine	4.48 × 10^−2^	0.1133	3.55	1.22 × 10^−1^	0.3237	3.91	2.35 × 10^−1^	0.3326	3.22

^a)^ Relative change, calculated by averaging the changes of each sample pair: (Treatment – Control)/Control) × 100.

^b)^ FDR, false discovery rate.

### 3.4 Effect of Silymarin on the Composition of Gut Microbiota

**Figure 5:**
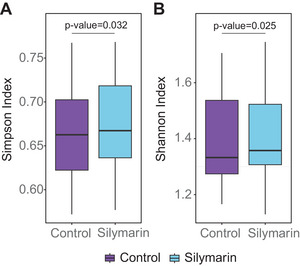
Alpha diversity at the level of order between control and silymarin‐treated samples after 24 h of incubation. The data represent mean values calculated across all donors (*n* = 20), each based on a single biological and technical replicate per donor. Error bars = SD: A) Simpson index; B) Shannon index.

The silymarin‐treated samples and the control group differed in α‐diversity of the overall microbiota at the order level, with the treatment samples showing statistically higher α‐diversity for both indices than the control group (Figure 5) (Simpson index: α_silymarin _= 0.674, α_control _= 0.667, *t* = 2.31, *p* = 0.032; Shannon index: *α*_silymarin _= 1.420, *α*_control _= 1.400, *t* = 2.43, *p* = 0.025). At the genus level, we found no statistically significant difference (Simpson index: *α*_silymarin _= 0.84, *α*_control _= 0.84, *t* = 0.64, *p* = 0.53; Shannon index: *α*_silymarin _= 2.64, *α*_control _= 2.63, *t* = 0.87, *p* = 0.40). These findings indicate a modest but significant increase in microbial diversity due to silymarin treatment.

### 3.5 Effect of Age on Silymarin Catabolism

**Figure 6:**
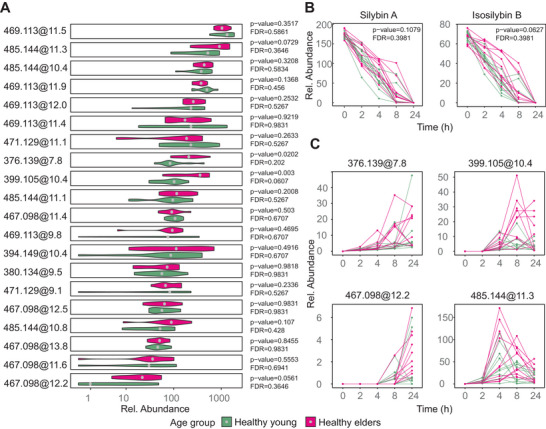
Effect of age on silymarin catabolism. A) area under the curve (AUC) of catabolites in the healthy young (age 12–45 years) and healthy elder subjects (age 70–80 years), The data represent mean values calculated across donors, divided into age groups (*n* = 10), each based on a single biological and technical replicate per donor; B) time course of catabolism of major silymarin components silybin A and isosilybin B; and C) time course of the formation and eventual degradation of major detected catabolites in individual subjects.

The study investigated the effect of age on the degradation of silymarin by dividing the donors into two groups: healthy young (aged 12–45 years, *n* = 10) and healthy elders (aged 70–80 years, *n* = 10). PERMANOVA showed marginally significant values for the effect of age on silymarin catabolism [*R^2^* = 0.117, *Pr* (> *F*)  =  0.055] when analysing the LC‐MS data. Univariate paired comparison based on AUC revealed that the unidentified metabolite 399.105@10.4 was higher in healthy elders (*p* = 0.0030, *q* = 0.0670). A few additional metabolites showed differences prior to the FDR correction, all of which were more abundant in healthy elder subjects (Figure 6A). The abundance of catabolite 399.105@10.4 was 2.5 times higher in the group of healthy elders compared to the healthy young group.

Marginal significance was found in the degradation of the original silymarin components. Isosilybin B was present more frequently in the healthy elders (*p* = 0.0630), which can be attributed to slower degradation. Figure 6A shows a violin plot depicting the differences between the age groups for all 20 identified silymarin catabolites, together with their corresponding *p* and *q* values. The time course of the above‐mentioned substances for each donor indicates differences in metabolic kinetics between individuals (Figure 6B).

### 3.6 Complex Interactions of Silymarin with Gut Microbiota through Data Fusion Analysis

**Figure 7:**
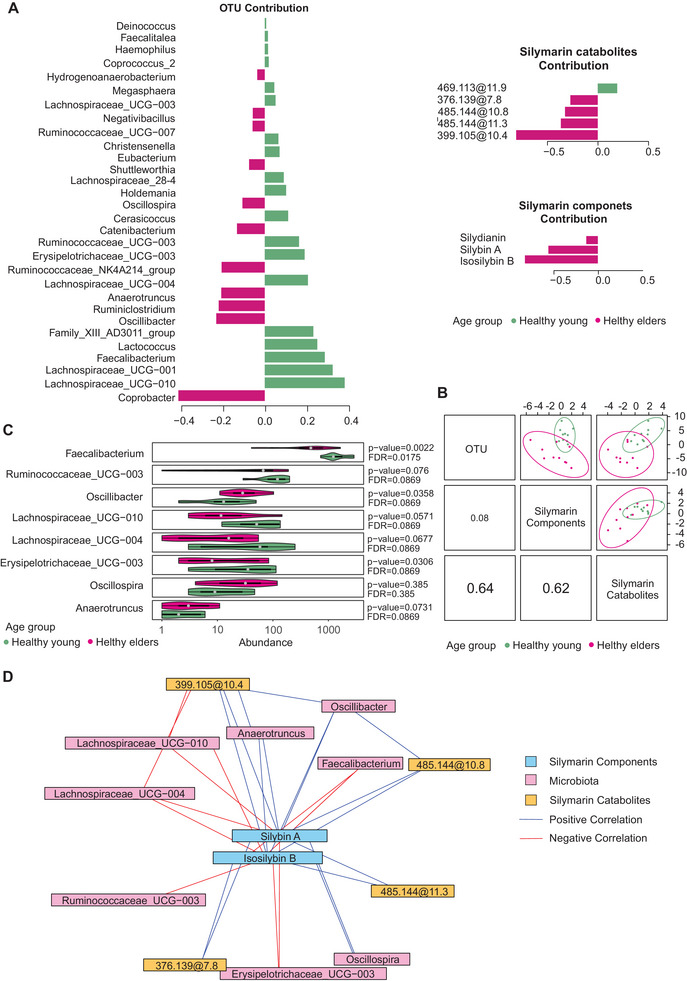
DIABLO integrative analysis of silymarin microbial catabolism and fecal microbiota discriminatory between healthy young and healthy elder groups. The data represent mean values calculated across donors, divided into age groups, each based on a single biological and technical replicate per donor. (A) Loading weights of the selected discriminant genera (OTUs), silymarin components and silymarin microbial catabolites; colors indicate the group in which the median relative abundance is a maximum, with values indicating the contribution to the first component, *n *= 10. (B) Matrix scatter plot showing the clustering of samples based on the first component in each dataset and the correlation between datasets, *n *= 10. (C) Difference in the abundance of microbial genera, included in the correlation network, between the healthy young (age 12–45 years) and healthy elder (age 70–80 years) groups, determined by independent Student's *t*‐test, *n *= 10. (D) Relevance network from DIABLO analysis depicting the correlation between microbiota, silymarin components, and catabolites; red lines represent positive correlations and blue lines represent negative correlations; the cutoff for the correlations was *r* = 0.6.

Having identified the catabolism of silymarin and its impact on the fecal metabolome, our objective was to investigate the interplay between the composition of the gut microbiota and silymarin catabolism in relation to age groups. To this end, we used the DIABLO algorithm on the mixOmics platform (Figure 7). One component was selected for the final model based on the lowest balanced error rate of 0.35 with a centroid distance metric. Subsequent parameter tuning included additional cross‐validation with fivefold cross‐validation (repeated 20 times), resulting in a final model with a cross‐validation error of approximately 0.28 containing 30 OTUs, three silymarin components from LC‐MS and five microbial silymarin catabolites from LC‐MS. The analysis revealed strong correlations between the gut microbial taxonomic profiles and the microbial silymarin catabolites (*r* = 0.64), as well as between the silymarin components and microbial catabolites (*r* = 0.62) (Figure 7B). Analysis at the genus level showed that healthy elders had a stronger association with certain bacterial genera, including *Coprobacter*, *Oscillibacter, Ruminoclostridium*, and *Anaerotruncus*. On the other hand, *Lachnospiraceae, Faecalibacterium*, and *Lactococcus* were more significant for the healthy young group. The parent components isosilybin B, silybin A, and silydianin were associated with the healthy elder group (Figure 7A). These findings are consistent with the results shown in the previous section, where they were hinted at using *t*‐tests (Figure 5). The contribution of the catabolite 399.105@10.4 to the healthy elder group was also confirmed. Conversely, the healthy young group was associated with catabolite 469.113@11.9. The contribution of each selected feature based on its loading weights is depicted in Figure 7A.

Correlation network analysis indicated interactions between silymarin, its microbial catabolites and the gut microbiota (Figure 7D). In particular, certain bacterial genera, including *Lachnospiracae* UCG‐10, *Lachnospiracae* UCG‐004, *Ruminococaceae* UCG‐003, *Erysipelotrichacae* UCG‐003, and *Faecalibacterium*, showed negative correlations with silybin A and isosilybin B, indicating their possible role in the degradation of these silymarin components. Notably, *Faecalibacterium* (*p* = 0.0022, *q* = 0.0175) and *Erysipelotrichacae* UCG‐003 (*p* = 0.0306, *q* = 0.0869) were found in higher abundance in healthy young individuals (Figure 7C), as revealed by independent *t*‐test. Conversely, *Oscillospira*, *Oscillibacter*, and *Anaerotruncus* were positively correlated with these silymarin components. *Oscillibacter* was found to be more abundant in healthy elder participants (*p* = 0.0358, *q* = 0.0869). This genus also showed a positive correlation with the presence of catabolite 399.105@10.4, high levels of which were typically found in the group of healthy elder individuals.

## 4 Discussion

Recent studies identified two major catabolites of a mixture of silybins A and B. These catabolites have the molecular formulas C_24_H_20_O_10_ and C_24_H_22_O_10_^[^ ^45^ ^]^: the first corresponds to demethylation of the methoxy group on the D‐ring of silybin B; and the second corresponds to the combination of this demethylation with reduction of the ether bridge on the D‐ring of silybin A between C11 and C12a to form methylene. These results are consistent with our experiment. The catabolites that we identified also include the above‐mentioned molecular formulas. The most abundant catabolite (469.113@11.5) corresponds to demethylation. Given its abundance and comparing the peak order by retention time with this mass in the above study, we predict its formation from silybin B. We also observed other reactions, such as demethylation coupled with dihydrogenation and dihydrogenation alone, both previously mentioned in in vitro^[^ ^15^, ^45^ ^]^ and in vivo^[^ ^46^ ^]^ studies. We also observed dehydroxylation alone, reported previously in the interventional study^[^ ^46^ ^]^ but not in vitro.^[^ ^15^, ^45^ ^]^ Dehydroxylation of flavonolignans may increase their hydrophobicity and improve passive diffusion across the epithelium.^[^ ^47^ ^]^ Even in this scenario, we anticipate that the dehydroxylated metabolites primarily originate from the most abundant constituents, silybin B and silybin A. Demethylation, dihydrogenation, dehydroxylations, and their combinations are common reactions occurring during the catabolism of dietary (poly)phenols such as ferulic acid,^[^ ^48^ ^]^ urolithins,^[^ ^49^ ^]^ anthocyanins,^[^ ^50^ ^]^ stilbenoids,^[^ ^51^ ^]^ or rutin.^[^ ^52^, ^53^ ^]^ In the case of silymarin, we have not observed the collapse of the structure through reactions such as ring fission, as seen in the catabolism of quercetin,^[^ ^53^ ^]^ rutin,^[^ ^52^ ^]^ or flavan‐3‐ols as catechin.^[^ ^53^, ^54^ ^]^ Instead, we observed the removal of peripheral substituents.

Numerous studies have shown that (poly)phenols can alter the composition of the gut microbiota by promoting the growth of beneficial bacteria and inhibiting the proliferation of pathogenic species.^[^ ^55^, ^56^ ^]^ These interactions may lead to changes in microbial α‐diversity.^[^ ^57^, ^58^ ^]^ Our study results show a modest yet statistically significant increase in α‐alpha diversity at the order level, with the Simpson index rising by 0.007 and the Shannon index by 0.020. In general, studies on animal models investigating the effects of (poly)phenols on gut microbiota have been inconclusive, with some observing a significant impact,^[^ ^57^ ^]^ while others did not.^[^ ^58^ ^]^ These variations may depend on the specific structure and dosage of the (poly)phenols used.

The impact of silymarin on microbiota could be influenced by certain limitations in our study, notably the sequencing technique and batch incubation approach, which may not accurately differentiate between live and dead bacterial cells, potentially masking differences. Additionally, dosage variations across studies can affect results. In our approach, we utilized a relatively low but diet‐relevant concentration of 50 µg mL^−1^, which we previously carefully calculated and reasoned.^[^ ^59^ ^]^ At this dosage, the likelihood of silymarin exhibiting antimicrobial activity and altering microbial populations in the human gut is low because it does not reach the commonly reported minimum inhibitory concentrations.^[^ ^60^, ^61^, ^62^ ^]^ However, as shown in our results, this concentration of silymarin leads to suppressed or slower fermentation and triggers changes in the fermentation pathways, reflected by increased monomeric carbohydrate levels (glucose, trehalose) and amino acids, and decreased production of their downstream products SCFAs (including BCFAs). While SCFAs are typically beneficial for gut pH balance, mucosal health, and inflammation reduction,^[^ ^63^, ^64^ ^]^ they can paradoxically rise in certain pathological conditions,^[^ ^65^ ^]^ such as in individuals with high BMI and diabetes.^[^ ^66^, ^67^ ^]^ This increase may be associated with changes in microbiota composition and enhanced energy extraction, which can be undesirable.^[^ ^68^ ^]^ Microbial imbalances are also key contributors to metabolic and liver diseases, including non‐alcoholic fatty liver disease.^[^ ^69^, ^70^ ^]^

The most expressed effect was seen in our study on BCFAs. These are derived from the fermentation of valine, leucine, and isoleucine. BCFAs can affect lipid metabolism and inflammatory responses in adipose tissue, playing a role in metabolic health.^[^ ^71^, ^72^ ^]^ Previous studies suggest that BCFA levels are elevated in individuals with higher BMI and age,^[^ ^71^ ^]^ and are linked to proteolytic fermentation, which produces harmful metabolites like ammonia and *p*‐cresol, particularly in older adults.^[^ ^71^, ^73^ ^]^ These associated metabolites may principally affect the viability of colonic epithelial cells or promote genomic DNA damage.^[^ ^73^ ^]^

Notably, silymarin had a stronger effect on fermentation in healthy elderly individuals compared to younger subjects. Additionally, in the elderly, it exhibited a different spectrum of catabolic products, suggesting potential age‐related differences in its activity. This underscores the need for well‐designed studies to explore silymarin's effects across age groups, as no clinical trials have yet stratified populations to assess this.

By suppressing fermentation, silymarin may help reduce excessive energy extraction, revealing a potential new mechanism of action. This effect was observed at low concentration, indicating that higher doses, such as those found in supplements, could lead to a more pronounced impact, warranting further investigation.

DIABLO analysis confirmed the previously reported differences in fecal microbial composition between healthy young and healthy elder subjects.^[^ ^74^, ^75^, ^76^, ^77^ ^]^ We have shown that these differences are associated with a change in silymarin degradation and catabolite production. Our study revealed a higher abundance of the genera *Lachnospiraceae* UCG‐010, *Lachnospiraceae* UCG‐004, *Ruminococcaceae* UCG‐003, and *Erysipelotrichaceae* UCG‐003 in the microbiome of healthy young donors. This is a confirmation of previously known differences between microbiota of young and elderly people. The *Lachnospiraceae* and *Ruminococcaceae* families are frequently associated with the production of SCFAs.^[^ ^78^ ^]^ In contrast, there is no direct evidence specifically addressing the role of the *Erysipelotrichaceae* family in SCFA production. However, studies have highlighted its potential impact on gut health and metabolic disorders.^[^ ^79^ ^]^ The higher abundance of these taxa of *Lachnospiraceae* and *Ruminococcaceae* family is linked to young age and better health outcomes. It is increased in subjects with lower total cholesterol levels.^[^ ^80^ ^]^ Higher abundances of *Lachnospiraceae* UCG‐010 and *Lachnospiraceae* UCG‐004 were also observed in individuals with higher intakes of certain other polyphenols.^[^ ^81^, ^82^ ^]^ Similarly, the genus *Ruminococcaceae* UCG‐003 is associated with a lower risk of the metabolic syndrome, dyslipidemia, and other cardiometabolic diseases.^[^ ^82^ ^]^ In our study, all these strains were also associated with faster degradation (lower area under the curve) of silybin A and isosilybin B. These genera were also associated with the formation of the catabolite 469.113@11.9, suggesting their involvement in the degradation of silybin A and isosilybin B via demethylation, leading to the formation of the catabolite 469.113@11.9.

The abundance of genera *Oscillibacter* and *Anaerotruncus* was in opposite associated with healthy elder donors. The genus *Oscillibacter* has been demonstrated to induce remission in ulcerative colitis patients following fecal microbiota transplantation.^[^ ^82^ ^]^ Furthermore, its levels decrease in individuals consuming a high‐fat diet, and this reduction is linked to the development of insulin resistance and gastrointestinal neoplasms.^[^ ^83^, ^84^ ^]^ While the genus *Oscillibacter* may have beneficial functions in healthy elder donors, the abundance of the genus *Anaerotruncus* appears to be linked to diets rich in saturated fats, obesity, and the development of certain pro‐inflammatory diseases.^[^ ^85^ ^]^ Based on the relevance network analysis, these genera are positively associated with the production of catabolite 399.105@10.4, which was increased by 2.5 times in healthy elder donors compared to the young donors. Our study determined the different fermentation profiles associated with different microbiomes of the young and elder subjects. This could have previously unknown implications for silymarin metabolism, potentially changing its metabolite profile and, consequently, its biological activities.

## 5 Conclusion

This study reveals novel, age‐dependent interactions between silymarin and the gut microbiota. Using a comprehensive multi‐omics approach and an ex vivo fermentation model, we observed that silymarin significantly suppresses fermentation. This effect was more pronounced in samples representing healthy elderly individuals, who also exhibited a distinct spectrum of silymarin catabolites linked to specific microbes like *Oscillibacter*. In contrast, younger subjects showed faster degradation of silymarin components associated with microbes such as *Faecalibacterium*.

These findings suggest a previously unrecognized mechanism through which silymarin may modulate the activity of gut microbiota, with potential implications for energy extraction, metabolic and liver health. By demonstrating that the gut microbiota's ability to metabolize silymarin varies with age, our study underscores the importance of personalized nutritional strategies that consider individual microbiome profiles.

This study advances the field by employing an innovative approach that integrates metabolomics, microbial sequencing, and silymarin catabolite analysis. Additionally, we demonstrate how age can influence the metabolism and potential efficacy of dietary polyphenols, a perspective not previously explored. Our findings open new pathways for age‐targeted dietary interventions and deepen our understanding of the complex relationships between diet, microbiota, and metabolic health.

## Conflict of Interest

The authors declare no conflict of interest.

## Author Contributions

J.H. and K.V. conceived, designed, and supervised the study and the manuscript writing. K.T. and V.Jar. performed the experiments and analyzed data. K.T. wrote the manuscript. P.M. performed LC‐MS analyzes and supervised data analyzes. A.M.B. collaborated with NMR analyses. O.C. and L.H. performed 16S rRNA analyses. V.Jan. and, partially, A.M.B. carried out the statistical analyses. P.K. and V.Jar. critically evaluated the manuscript. All authors read and approved the version submitted.

## Supporting information



Supporting Information

## Data Availability

Data available on request from the authors.
