# Supplementary material for: Mutual Interactions of Silymarin and Colon Microbiota in Healthy Young and Healthy Elder Subjects
Source: Mol Nutr Food Res. 2024 Oct 30;68(22):2400500. doi: 10.1002/mnfr.202400500 (PMC11605779; doi:10.1002/mnfr.202400500)
Supplement: Supplementary file 1 — Supporting Information [file MNFR-68-2400500-s001.docx]

**Supplementary Material**

Mutual Interactions of Silymarin and Colon Microbiota in Healthy Young and Healthy Elder Subjects

Katerina Tomisova, Veronika Jarosova, Petr Marsik, Anna Mascellani Bergo, Ondrej Cinek, Lucie Hlinakova, Pavel Kloucek, Vaclav Janousek, Kateřina Valentová, Jaroslav Havlik^1^

**List of tables**

| Table number | Title | Page |
| --- | --- | --- |
| Table S1 | Demographic data of human stool donors | 2 |
| Table S2 | Table of all metabolites quantified by 1H NMR and their corresponding AUC concentrations in fecal fermentation samples (µM/ml/24 hours) | 3 |

**Table S1.** Demographic data of human stool donors

| **Donor name** | **Age (years)** | **BMI** | **Sex** | **Age group** |
| --- | --- | --- | --- | --- |
| D01 | 12 | 16.22 | MALE | Healthy young |
| D02 | 42 | 25.98 | MALE | Healthy young |
| D03 | 80 | 24.74 | FEMALE | Healthy elders |
| D04 | 75 | 24.73 | FEMALE | Healthy elders |
| D05 | 76 | 25.78 | FEMALE | Healthy elders |
| D06 | 76 | 26.59 | MALE | Healthy elders |
| D07 | 75 | 24.02 | FEMALE | Healthy elders |
| D08 | 75 | 31.07 | MALE | Healthy elders |
| D09 | 45 | 21.55 | MALE | Healthy young |
| D10 | 41 | 20.94 | FEMALE | Healthy young |
| D11 | 28 | 24.46 | FEMALE | Healthy young |
| D12 | 26 | 23.41 | MALE | Healthy young |
| D13 | 30 | 22.86 | FEMALE | Healthy young |
| D14 | 25 | 30.68 | FEMALE | Healthy young |
| D15 | 32 | 28.65 | FEMALE | Healthy young |
| D16 | 24 | 25.54 | FEMALE | Healthy young |
| D17 | 70 | 28.13 | FEMALE | Healthy elders |
| D18 | 72 | 26.30 | MALE | Healthy elders |
| D19 | 74 | 27.85 | FEMALE | Healthy elders |
| D20 | 71 | 24.69 | MALE | Healthy elders |

**Table S2.** Table of all metabolites quantified by 1H NMR and their corresponding AUC concentrations in fecal fermentation samples (µM/ml/24 hours)

| **Compund / Donor** | **D01** | **D02** | **D03** | **D04** | **D05** | **D06** | **D07** | **D08** | **D09** | **D10** | **D11** | **D12** | **D13** | **D14** | **D15** | **D16** | **D17** | **D18** | **D19** | **D20** |
| --- | --- | --- | --- | --- | --- | --- | --- | --- | --- | --- | --- | --- | --- | --- | --- | --- | --- | --- | --- | --- |
| 5-Aminopentanoate | 6400 | 4017 | 4271 | 3754 | 13698 | 1701 | 3461 | 1238 | 4771 | 1649 | 7014 | 1463 | 10600 | 4903 | 1899 | 2905 | 9014 | 11016 | 7692 | 6960 |
| Acetate | 830714 | 748839 | 620304 | 430525 | 571438 | 648085 | 787256 | 701536 | 629665 | 762397 | 722633 | 614190 | 752801 | 629048 | 653564 | 713168 | 675648 | 724215 | 596320 | 540371 |
| Alanine | 18130 | 18127 | 20418 | 19564 | 12708 | 10630 | 8328 | 10033 | 10603 | 13593 | 9882 | 6667 | 33063 | 30517 | 11074 | 9930 | 18340 | 23194 | 20693 | 19191 |
| Asparagine | 428 | 637 | 496 | 688 | 428 | 428 | 489 | 428 | 507 | 589 | 428 | 428 | 667 | 553 | 428 | 477 | 428 | 428 | 479 | 428 |
| Butyrate | 142091 | 151173 | 107720 | 87716 | 134544 | 129570 | 162427 | 114000 | 154271 | 134374 | 110898 | 127451 | 151311 | 192368 | 135959 | 174797 | 156349 | 142279 | 116198 | 123383 |
| Cadaverine | 62 | 374 | 465 | 218 | 2521 | 3904 | 62 | 924 | 2553 | 953 | 3991 | 3402 | 981 | 1579 | 242 | 4001 | 216 | 400 | 62 | 151 |
| Cysteine | 12310 | 12310 | 12310 | 12310 | 12310 | 12310 | 12310 | 12310 | 12310 | 12310 | 12310 | 12310 | 52841 | 66609 | 12310 | 12310 | 12310 | 12310 | 12310 | 12310 |
| Cystine | 767 | 1123 | 1317 | 230 | 230 | 230 | 230 | 462 | 2055 | 2273 | 1527 | 717 | 18968 | 30161 | 1417 | 230 | 426 | 230 | 1134 | 1091 |
| Ethanol | 37684 | 10641 | 22146 | 95073 | 45595 | 55927 | 49216 | 35711 | 19089 | 35877 | 48181 | 67689 | 73938 | 36950 | 25978 | 34001 | 14280 | 33438 | 35049 | 25884 |
| Glucose | 20850 | 22240 | 47622 | 55133 | 31224 | 29477 | 21552 | 28068 | 33039 | 29090 | 41525 | 41504 | 31005 | 19339 | 34718 | 26289 | 45856 | 51615 | 43959 | 49554 |
| Glutamate | 7705 | 6123 | 8843 | 21923 | 8003 | 6094 | 9078 | 10880 | 9034 | 8082 | 9222 | 7261 | 8807 | 10453 | 9644 | 10049 | 10815 | 19982 | 10125 | 11527 |
| Glycine | 1460 | 1907 | 3179 | 3272 | 3183 | 3661 | 3154 | 1783 | 2006 | 1961 | 2443 | 2080 | 3588 | 3218 | 2341 | 2698 | 1845 | 3965 | 2591 | 3560 |
| Isobutyrate | 14894 | 18825 | 5988 | 9461 | 12913 | 15470 | 26291 | 15789 | 15615 | 13623 | 13918 | 16640 | 1506 | 2895 | 15631 | 16643 | 9254 | 7465 | 10817 | 7103 |
| Isoleucine | 8986 | 6699 | 11659 | 9475 | 10474 | 9891 | 4519 | 7375 | 8157 | 9134 | 7478 | 4403 | 18095 | 16909 | 8018 | 8002 | 11674 | 14760 | 10055 | 12309 |
| Isovalerate | 15237 | 18775 | 6197 | 9707 | 12035 | 16536 | 26731 | 17253 | 17359 | 14773 | 16091 | 16627 | 1501 | 2979 | 16189 | 16599 | 9503 | 8221 | 10038 | 7693 |
| Lactate | 6607 | 9629 | 4358 | 6173 | 85443 | 21495 | 9154 | 6028 | 9002 | 7394 | 15955 | 7195 | 76690 | 16099 | 6684 | 10337 | 7647 | 29567 | 4540 | 47122 |
| Leucine | 14785 | 12972 | 21532 | 18150 | 17593 | 15781 | 8875 | 14111 | 15782 | 16237 | 11875 | 9575 | 29271 | 25836 | 14883 | 14811 | 20813 | 24433 | 20077 | 22483 |
| Lysine | 4735 | 3216 | 4219 | 7376 | 5191 | 2743 | 3264 | 2371 | 4067 | 3405 | 2539 | 2146 | 1294 | 208 | 2923 | 2528 | 4651 | 4318 | 4788 | 2179 |
| Maltose | 759 | 4697 | 626 | 4699 | 626 | 626 | 626 | 626 | 3708 | 1653 | 1923 | 3768 | 1184 | 3868 | 844 | 626 | 767 | 626 | 2744 | 626 |
| Methanol | 26405 | 18906 | 29691 | 28827 | 23562 | 24229 | 24081 | 16409 | 21339 | 20318 | 24857 | 25634 | 26273 | 24117 | 34734 | 34872 | 16644 | 19141 | 19761 | 13657 |
| Methionine | 3331 | 2030 | 3767 | 4984 | 1356 | 2408 | 1470 | 4092 | 2144 | 4332 | 4687 | 2056 | 7958 | 5475 | 2489 | 1797 | 2202 | 5478 | 2954 | 4854 |
| Phenol | 4354 | 290 | 290 | 290 | 1592 | 793 | 1922 | 290 | 483 | 2313 | 1078 | 290 | 290 | 290 | 290 | 706 | 1274 | 290 | 290 | 290 |
| Phenylacetic Acid | 5814 | 8962 | 7743 | 4625 | 5067 | 7608 | 6975 | 4929 | 7372 | 6441 | 7278 | 7567 | 495 | 1002 | 6143 | 5740 | 5850 | 6375 | 4294 | 4263 |
| Phenylalanine | 8078 | 6278 | 6122 | 9295 | 8874 | 7805 | 9136 | 9389 | 7603 | 7493 | 5000 | 4491 | 15343 | 12843 | 8287 | 8524 | 8416 | 9389 | 9917 | 10135 |
| Proline | 3002 | 889 | 537 | 5241 | 1988 | 537 | 537 | 1266 | 1350 | 1821 | 1765 | 537 | 13759 | 18179 | 2726 | 1854 | 537 | 2566 | 1515 | 1649 |
| Propionate | 127996 | 175247 | 107708 | 41901 | 205411 | 245603 | 288713 | 143337 | 170194 | 186037 | 247583 | 242356 | 72685 | 127426 | 134778 | 152769 | 106257 | 78729 | 150338 | 155311 |
| Pyroglutamate | 3027 | 3511 | 6924 | 10307 | 3121 | 3101 | 3272 | 6094 | 3694 | 3616 | 6441 | 4678 | 3920 | 4194 | 6379 | 3816 | 4131 | 9222 | 5945 | 6082 |
| Serine | 885 | 250 | 1567 | 250 | 250 | 583 | 250 | 250 | 250 | 722 | 250 | 250 | 2287 | 781 | 800 | 722 | 250 | 1011 | 250 | 250 |
| Succinate | 39799 | 1554 | 66623 | 75742 | 12995 | 2234 | 9567 | 47895 | 33870 | 6105 | 3432 | 9580 | 44132 | 48393 | 38805 | 12048 | 25993 | 35233 | 558 | 5371 |
| Threonine | 1756 | 1796 | 1947 | 2415 | 1256 | 1460 | 1097 | 1622 | 1593 | 1670 | 2138 | 1215 | 3350 | 2414 | 2237 | 1617 | 1779 | 3543 | 2399 | 2081 |
| Trehalose | 2276 | 3888 | 3832 | 4205 | 2962 | 2424 | 1370 | 3347 | 3673 | 3878 | 3813 | 3290 | 3990 | 3295 | 3406 | 1547 | 3426 | 4229 | 3889 | 2183 |
| Tryptophan | 824 | 724 | 877 | 1822 | 985 | 847 | 625 | 911 | 842 | 830 | 895 | 830 | 2020 | 1397 | 792 | 621 | 832 | 905 | 841 | 907 |
| Tyrosine | 1514 | 1349 | 1492 | 3495 | 1371 | 1472 | 1291 | 3604 | 2131 | 1584 | 1691 | 1323 | 3000 | 5236 | 3270 | 2470 | 2604 | 4327 | 3396 | 3573 |
| Valerate | 26964 | 45204 | 29479 | 18667 | 14567 | 50746 | 43725 | 42371 | 43762 | 43228 | 49019 | 35265 | 1963 | 17821 | 41285 | 34019 | 26314 | 20900 | 32516 | 35547 |
| Valine | 14319 | 12063 | 20344 | 16334 | 15385 | 14864 | 8613 | 12757 | 12743 | 14376 | 10911 | 8737 | 26463 | 24883 | 12799 | 13245 | 18567 | 22821 | 16486 | 18096 |
